# MethVisual - visualization and exploratory statistical analysis of DNA methylation profiles from bisulfite sequencing

**DOI:** 10.1186/1756-0500-3-337

**Published:** 2010-12-15

**Authors:** Arie Zackay, Christine Steinhoff

**Affiliations:** 1 Department of Computational Biology, Max Planck Institute for Molecular Genetics, Ihnestr 73, 14195 Berlin, Germany; 2 Dept. Microbiology and Molecular Genetics, IMRIC, Hebrew University-Hadassah Medical School, Jerusalem, Israel

## Abstract

**Background:**

Exploration of DNA methylation and its impact on various regulatory mechanisms has become a very active field of research. Simultaneously there is an arising need for tools to process and analyse the data together with statistical investigation and visualisation.

**Findings:**

MethVisual is a new application that enables exploratory analysis and intuitive visualization of DNA methylation data as is typically generated by bisulfite sequencing. The package allows the import of DNA methylation sequences, aligns them and performs quality control comparison. It comprises basic analysis steps as lollipop visualization, co-occurrence display of methylation of neighbouring and distant CpG sites, summary statistics on methylation status, clustering and correspondence analysis. The package has been developed for methylation data but can be also used for other data types for which binary coding can be inferred. The application of the package, as well as a comparison to existing DNA methylation analysis tools and its workflow based on two datasets is presented in this paper.

**Conclusions:**

The R package MethVisual offers various analysis procedures for data that can be binarized, in particular for bisulfite sequenced methylation data. R/Bioconductor has become one of the most important environments for statistical analysis of various types of biological and medical data. Therefore, any data analysis within R that allows the integration of various data types as provided from different technological platforms is convenient. It is the first and so far the only specific package for DNA methylation analysis, in particular for bisulfite sequenced data available in R/Bioconductor enviroment. The package is available for free at http://methvisual.molgen.mpg.de/ and from the Bioconductor Consortium http://www.bioconductor.org.

## Findings

### Motivation

DNA Methylation is a biochemical modification of DNA which occurs in vertebrates almost exclusively at CpG sites, e.g. a methyl group is added at the 5' C position of cytosines. Recently, exploration of DNA methylation and its impact on various regulatory mechanisms has become a very active field of research. Specific DNA methylation patterns and/or their impact on a number of regulatory processes have been described for cancer [1,2], silencing of repetitive elements [3-5], ageing, development and embryonic stem cell profiling [6-8], correlation with chromatin remodeling, X chromosome inactivation [9], RNA interference [10]; imprinting [11,12], tissue specific expression profiles [13] and evolutionary mutation processes [14]. The variety of regulatory mechanisms for which involvement of methylation has been reported demonstrates its high impact. Furthermore, recent results suggest a very close functional relationship between DNA methylation and histone modification [15]. Thus, in combination with other epigenetic events, DNA methylation might have an important regulatory impact.

While a number of technologies for quantitative and qualitative analysis of CpG methylation have been published over the last years, the most accurate experimental procedures are still based on bisulfite treatment followed by conversion of non methylated cytosines to uracil and sequencing. Analyzing this kind of data is complicated. Several steps, like alignment of bisulfite treated sequence to reference sequence, detection of low conversion rates of C to T in the bisulfite treatment or the conversion process and quality control, are necessary before actually extracting methylation profiles for further statistical analysis. However, this procedure is a prerequisite for the investigation of functionality of DNA methylation. Tools that allow appropriate processing of this kind of data are needed and currently being developed with different focuses [16-21]. The exploration of combinatorially acting factors for the determination of epigenetic features is a field of current research [22].

Bisulfite based DNA methylation data can be produced in a high throughput fashion and on the basis of single gene investigation. While high throughput DNA methylation profiling is a very new technological application and to date only few datasets are available single gene investigations are well established and have been proven as a gold standard in the field of epigenetics research [23,24]. Computationally processing of both kinds of data will be an importent ability in the future. This is because high throughput data will serve as a first step to identify candidate regions that will later be verified independently in single gene approaches. In this application we will focus on the single gene approaches, although in principle high throughput data can be processed and the package is open for further development in terms of more efficient analysis of high throughput data. In future versions one would allow for the assessment of both single gene investigations and high throughput data. Actually, Bioconductor offers a number of packages for analyzing high throughput data, like the SNPchip Package for SNP chip analysis [25]. In fact, SNPchip has been used for methylation analysis [26]. Using packages that are capable for analyzing large datasets in combination with statistical analysis and visualization implemented in MethVisual could be a powerful strategy for the analysis of DNA methylation data.

Over the last decade the open source and open development software platform R/Bioconductor has evolved and nowadays displays one of the most important software platforms for statistical investigations in biological and medical informatics. Thus, any package that is added to this software pool has the advantage that it can be easily combined and integratively used with other existing applications. In doing so, there is no need for performing analyses using separate tools within one investigative pipeline. Currently, there is no package in the R/Bioconductor environment that allows for preprocessing, statistical analysis and visualization of DNA methylation data apart from MethVisual. However, there are few software tools outside R/Bioconductor that allow for bisulfite sequenced data analysis: MethTools [18] comprises a set of perl scripts and allows for estimation of systematic experimental errors and display of methylation data. A tool for comprehensive statistical investigation is not included and the program requires pre-aligned sequences as input. The Java program BiQ-Analyzer [17] overlaps with respect to alignment, quality check and basic plotting functions with the proposed MethVisual tool. Further statistical investigation and the integrative analysis of covariate data (for example patient information on tumor staging, survival, etc) are implemented in BIQ-Analyzer but cannot be performed within that tool. The most recent program, QUMA [16] is an interactive web based tool, also allowing testing independence of each CpG site methylation between two groups (Fisher's exact test) or of entire sets of CpG sites (Mann-Whitney U test).

Here we present a new R/Bioconductor package that is the first DNA methylation processing tool within R/Bioconductor. As we show, it is comparable with tools such as BIQAnalyzer and QUMA; we have added some exploratory statistics features, for example, principle component analysis related methods. Even though MethVisual has been developed for DNA methylation data it can be used in a straightforward manner for various other types of data that can be in a first step discretized and then binarised for example by indicator matrix coding. Another possibility to apply the package is data concerning the binding of specific transcription factors or histone modification.

### MethVisual package description

**Figure 1:**
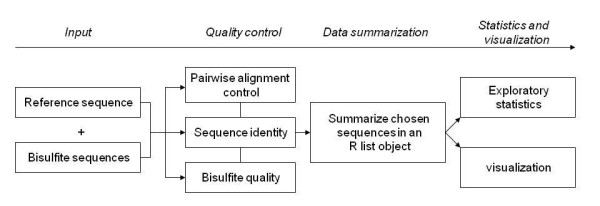
**Workflow for MethVisual**. The figure visualizes the analysis steps using MethVisual starting from sequencer output.

The MethVisual R package performs alignment, quality control, visualization and statistical analysis. A workflow of the software demonstrating data processing steps is shown in Figure 1. The individual processing steps are introduced here. For closer details we refer to the vignette [27].

### Reading Sequences (Reference Sequence and Bisulfite Sequence)

The input data can be provided as multiple or separate FASTA files [28] or gff files [29]. Furthermore, the reference DNA sequence, which is used as a reference to determine the genomic location of DNA methylation, has to be provided by the user in FASTA format. Due to the fact that for some experimental methods the data is continuous we implemented a function for the discretization of continuous data into binary coding which is based on thresholding. This function can be used for various kinds of data as well, for example if one wishes to discretize expression data based only on thresholding. More sophisticated discretized expression data might be processed by applying other tools within R/Bioconductor, for example POE [30].

### Quality Control

The alignment control (AC) procedure comprises a comparison to the reference sequence and is performed to prevent false alignment during further analysis. False alignment can occur for three reasons: sequences that are 1) reversed, 2) complement or 3) reversed-complement to the reference sequence. MethVisual compares the score result computed by the pairwise Needleman-Wunsch Algorithm for global alignment implemented in the Biostrings package [31] and selects the alignment variant with the highest score among these three possibilities mentioned above for each bisulfite sequence involved. The alignment score is calculated due to DNA alphabet substitution matrix (IUPAC code). Because of the special characteristic of the bisulfite sequences the substitution matrix tolerates the alignment of T in bisulfite sequences to C in reference sequence.

Bisulfite conversion, which involves conversion of non methylated cytosines (Cs) to uracil (Us) upon bisulfite treatment and subsequent amplification, can be an incomplete process where non methylated Cs have not been converted. MethVisual measures the bisulfite treatment quality by calculating this conversion ratio which is defined as the ratio between the number of unconverted Cs within non CpG sites and the sum of all Cs outside CpG sites. MethVisual computes sequence identity rate of the bisulfite sequences to the reference sequence by calculating the nucleotide matches and mismatches in a local pairwise alignment. The usage of this procedure is motivated by Kumaki et al. [4] restricting the comparison of sequenced sample - thus the bisulfite treated clone sequence - versus reference sequence to the three bases A, G and T.

### Data Summary

In order to analyze and visualize the DNA methylation pattern we need to summarize data in the next step. We save the data information consisting of: (i) bisulfite sequence name, (ii) methylation matrix, e.g. clone sequences times CpG positions binary coded matrix for indications of methylation and non methylation, (iii) positions of CpG sites in the reference sequence and start and end position of the pairwise alignments as an R object for further analysis.

### Statistics and Visualization

For straightforward visualization of the processed methylation profiles we implemented lollipop plots as well as neighbouring and distant co-occurrence display. Neighbouring co-occurrence display shows the common methylation patterns between neighbouring CpG sites. This can be displayed as percentage or correlation of sharing methylation events between two neighbouring CpG sites. Frequently one would also like to know whether any two non-directly neighbouring CpG sites display a relatively high or low common methylation pattern across experiments. Therefore, we also included the new option to display the co-occurrence between any two CpGs under study. In fact, for structure analysis of Dnmts Jia et al. [32] showed that there is a periodicity for which the 10 bps apart located CpG is preferentially methylated. Analyzing the functional relevance of methylation patterns depending on their relative position is an interesting open question.

We also included a number of statistical applications. These options include (i) percentage of methylated and non methylated sites in sets of experiments (ii) testing for independence of each CpG site between two groups (Fisher's exact test) or (iii) of entire sets of CpG sites (Mann-Whitney U test). Furthermore, (iv) we provide a hierarchical bi-clustering option based on the quality-checked methylation data matrix. Due to the fact that we analyze binarized rather than continuous data the default option between two binarized methylation patterns is the Hamming distance rather than the euclidean distance. One has to keep in mind, that this method does not take into account the genomic ordering of CpG sites.

**Figure 2:**
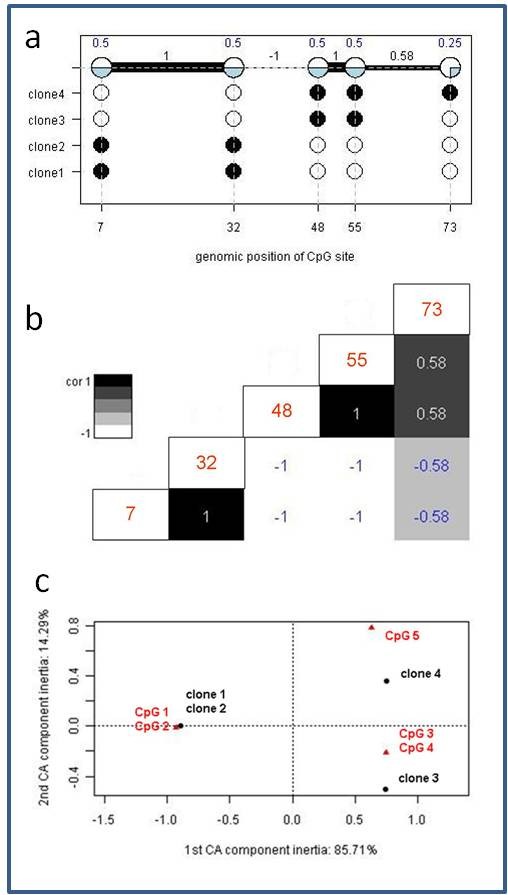
**MethVisual example**. (a) Lollipop displays for 4 binarized methylation profiles in the genomic context are shown. Filled dots refer to methylated sites, empty dots to non methylated sites. Each clone of the experiment is displayed separately according to its methylation status. Lengths of the connecting lines correspond to relative genomic distance between CpG sites. A summary plot is given in the upper part of the figure. The percentage of filling of each dot corresponds to the percentage of clones that showed methylation of the respective CpG site. Furthermore, in the summary plot, the thickness of the connecting lines between each two CpG sites refers to the correlation of methylation between the two neighbouring CpG sites. (b) Example of distant co-occurrence plot. CpG sites are displayed in a matrix. Each pairwise comparison, e.g. neighbouring and distant, leads to a correlation value that is displayed in the matrix. Correlation is color coded and the color coding bar is given beside the graph. The numbers in the diagonal give the genomic position of each displayed CpG site (c) Example of correspondence analysis plot. The first two components of correspondence plot of co-occurrence are shown. Red triangles refer to the position of CpG sites, black bullets to clones under study. At the x and y axes correspondence component inertia of 85.71% and 14.29% are given.

Furthermore, we implemented correspondence analysis [33] for the intuitive visualization of the methylation matrix. This method allows for studying associations between CpG sites and subclones. Briefly, correspondence analysis is applied to a two way table which in our case are DNA methylation values of CpG sites measured in clones. The analysis is used for describing correspondence between columns (here CpG sites) and rows (here experiments/clones). First, we describe how to read a given display as for example shown in Figure 2c. In a second step we briefly describe the mathematical basis. However, for closer detail we refer to [34,35].

The basis for a correspondence analysis display is a data matrix providing a measurement for each clone in each CpG position. Applying correspondence analysis this matrix is displayed on a 2-dimensional plane as is shown in Figure 2c. The figure shows all clones and all CpG positions in one figure. It can be read in the following way: Similarity between each two points is given by the angle between the two points/objects. Objects (clones, CpG sites) with similar correlations are clustered together resulting in small angles, whereas dissimilar objects are separated from each other (large angle, e.g. different quadrants). Furthermore it holds, that the larger the vector length the higher the information content. In Figure  2c, each point, i.e. CpG site (red triangle) or clone (black dot), marks the direction and distance of a vector originating from the centroid. The appearance of vectors in the same quadrants and a closer angle distance between vectors reflects their relative association. Following the example, CpG sites 1 and 2 and clone 1 and 2 show a small angle. That means clone 1 and 2 are mainly determined by their CpG profile in position 1 and 2. In fact, exactly in these positions the clones are methylated. This feature distinguishes them from the other two clones. While correspondence analysis allows us to visualize associations in complex matrices, it should be noted that there is no threshold to decide whether an association is strong or weak, the vectors describe relative associations, i.e. stronger or weaker.

Originally, the method was described by Berzerci [36]. In biological data analysis context, correspondence analysis has been used for the first time for the analysis of microarray data in [35]. Correspondence analysis processes cross-tabulations of categorical data (contingency tables) by projecting the data vectors on the directions maximizing the total chi-square distance instead of the total variance, used for singular value decomposition and continuous data. Theoretically speaking, rotating the high dimensional data, each data point is projected onto a 2-dimensional planar in a way that maximal variance can be seen according to the first and second dimension. In our application, this allows for studying associations between clones and CpG sites.

### Computational processes

All calculations have been performed using R 2.11.0 version and Bioconductor 2.6 which was the latest versions at the time of writing the paper under Windows and Unix systems. The package MethVisual is currently available under Bioconductor 2.6 and has been checked under R version R 2.11.0. Any calculations within BIQAnalyzer and QUMA have been performed with default settings.

### Demonstration of pipeline

We produced a very simple methylation profile, consisting of four clones measuring 5 CpG sites at hypothetical basepair positions 7, 32, 48, 55 and 73. While two clones show identical methylation profiles, namely being methylated at CpG sites 1 and 2, and otherwise are non methylated, one clone is methylated at CpG sites 3 and 4, whereas for another clone methylation has been determined for CpG sites 3, 4, and 5. First, we demonstrate visualization based on the example data. Three important figures are summarized in an overview representation in Figure 2.

In Figure 2a the summary display at the top of the figure does not only display the relative amount of methylated clones for each CpG site (above the circles), but also shows the correlation between neighbouring CpG sites. Correlation can be visualized as the relative amount of shared methylation patterns or as a correlation coefficient using Hamming distance. CpG sites 1 and 2 are perfectly correlated, they are either both methylated (clones 3 and 4) or both unmethylated (clones 1 and 2). CpG sites 2 and 3 are perfectly anti-correlated. This means, if one site is methylated the other one always is not and vice versa. However, correlation between distant CpG sites, as for example CpG site 1 and 3 - which should be the same as CpG sites 2 and 3 - are more difficult to see. This can be omitted by using a matrix display as is provided by the function matrixSNP. An example is shown in Figure 2b. Here, a comprehensive graphical representation has been produced applying lollipop plots combined with neighbouring and distant co-occurrence. For space reasons we omitted the lollipop display which is already displayed (even though not equidistant) in the figure part above (Figure 2a) and only show the distant co-occurrences. Basically, this is a colour coded distance matrix which in this intuitive case can easily be compared with the summary plot in Figure 2a. In more complicated data structures the colour coded matrix might be easier to access and thus is a more appropriate display.

In Figure 2c we apply correspondence analysis [33]. This allows us to study associations between CpG sites 1-5 and subclones 1-4. Objects (CpG sites, clones) with similar correlations cluster together resulting in small angles, whereas dissimilar objects are separated from each other by a large angle, e.g. different quadrants. In this very simple example, one expects, that clones 1 and 2 should group together and CpG sites 1 and 2 should display a small angle to clones 1 and 2 because their methylation characterizes the two clones. On the other hand, clones 3 and 4 should separate strongly from clones 1 and 2 since they show opposite patterns and are mostly determined by their methylation pattern in CpG site 3, 4 and 5. In fact inertia of the first correspondence component is 85.71% whereas the second comprises 14.29%. Thus the two dimensions displayed in Figure 2c explain a large majority of variety captured in this data example.

### DNA methylation data processing: artificial data

BiQ-Analyzer offers the user a tutorial dataset. We use the tutorial datasets that includes one reference sequence (223 bps) and ten artificial clone sequences (seqA, seqB,.., seqJ) in FASTA format corresponding to the reference sequence. Basically, in this subsection we want to demonstrate that MethVisual gives similar results as BIQAnalyzer and QUMA. We would like to stress that we do not aim at providing a completely new tool but rather the first Bioconductor tool that can be used integratively with existing statistical applications in R but have added some new features that are useful for visualizing DNA methylation data and are not present in other DNA methylation tools.

We processed the BIQAnalyzer dataset with BIQAnalyzer and MethVisual R package in parallel to compare alignment and quality control. Analyzing the data in MethVisual showed how the program deals with problematic data in the context of false alignments, low clone sequence quality and problems with cytosine conversion due to bad bisulfite treatment. Analyzing the data in MethVisual and comparing the results with those in BIQAnalyzer confirmed the MethVisual alignment and conversion correctness as shown in additional file 1. The identity control value calculated by MethVisual and BIQAnalyzer differ in 3 out of 10 cases. This is due to different alignment calculations.

**Figure 3:**
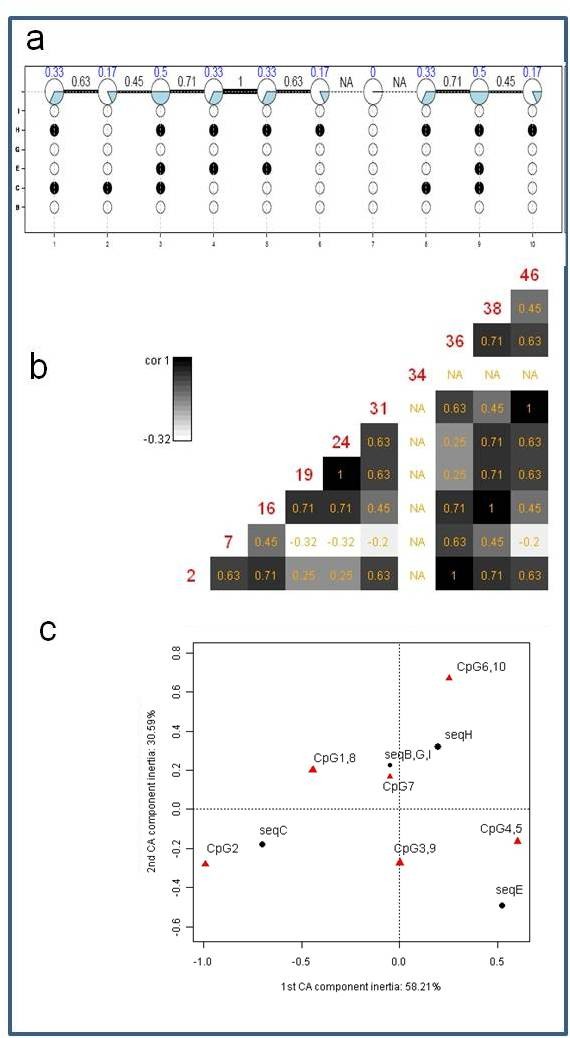
**MethVisual BIQAnalyzer study data**. (a) Lollipop displays for reduced BIQ Analyzer study dataset as in figure 2a (b) BIQ Analyzer study dataset distant co-occurrence plot as in figure 2b (c) BIQ Analyzer study dataset correspondence analysis plot as in figure 2c

**Figure 4:**
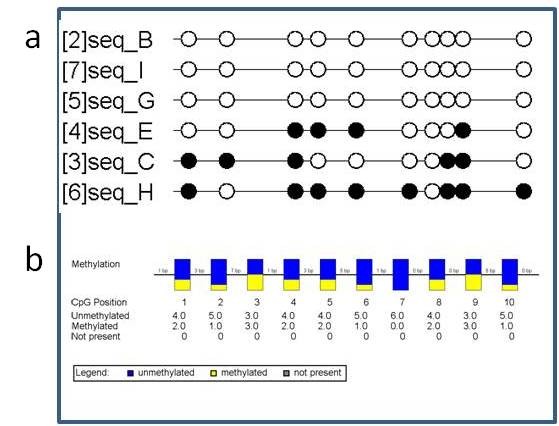
**BIQAnalyzer study data**. (a) The reduced BIQAnalyzer tutorial dataset was processed within BIQAnalyzer with default settings and a lollipop display was produced. Filled (methylated) and empty (non methylated) circles mark relative genomic positions of CpG sites under study. Each row shows one clone that was analyzed, providing the clone name beside (left). The line break was produced automatically by the tool. (b) The reduced BIQAnalyzer tutorial dataset was processed within BIQAnalyzer with default settings and a summary display was produced. The boxes display a summary of relative amount of clones being methylated (yellow) or non methylated (blue) at a specific CpG site. Between neighbouring CpG sites genomic distances (not correlation) are displayed. The rows below the boxes show the genomic basepair position, the absolute amount of methylated, non methylated and not present clones for each site.

**Figure 5:**
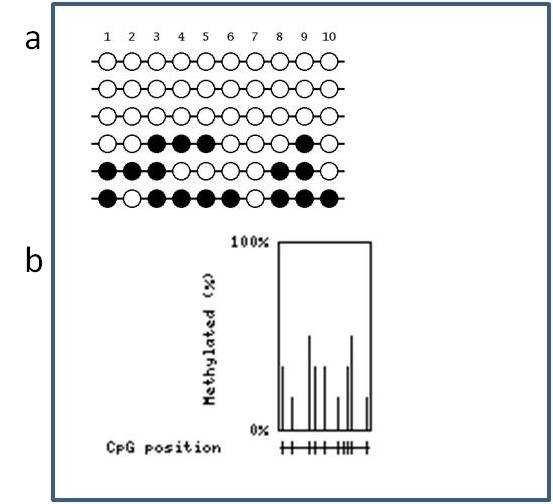
**QUMA BIQAnalyzer study data**. (a) The reduced BIQAnalyzer tutorial dataset was processed within QUMA and a lollipop display with equidistant CpG sites was produced. Filled (methylated) and empty (non methylated) circles mark relative genomic positions of CpG sites under study. (b) The reduced BIQAnalyzer tutorial dataset was processed within QUMA and a display showing the amount of methylation for each CpG site is shown as it is provided by QUMA.

In order to clearly represent the results we restrict our comparison and visualization to the central 10 CpGs located at relative genomic positions 2, 7, 16, 19, 24, 31, 34, 36, 38 and 46. As for the data above we produce an overview figure showing lollipop plot with neighbouring correlations (Figure 3a), distant co-occurrence plot (Figure 3b) and correspondence analysis plot (Figure 3c). In Figure 3a we chose an equidistant display for space reasons. Figure 4 shows the respective overview plots as provided from the BIQ Analyzer software, whereas Figure 5 displays outputs from QUMA. Comparing displays of Figure 3a and Figure 4 there is much information directly accessible that is not included in the BIQAnalyzer display. For example, neighbouring correlations can be seen, e.g. CpG sites 4 and 5 are perfectly correlated. At the same time the percentage of methylated clones which the correlation is based on can be read as 33%. In BIQAnalyzer two plots have to be generated to see clonewise methylation and correlation characteristics. We further show a display of distant correlation produced by MethVisual (Figure 2b). For example, CpG sites 3 and 9 at basepair positions 16 and 38 are perfectly correlated based on the methylation of three clones but interrupted by 5 CpG sites. Thus, looking only at the Lollipop plot as shown in Figure  3a, distant correlation features are harder to see.

QUMA is an interactive web based tool for DNA methylation analysis [16]. The input data can be provided in FASTA format, GenBank3 or as plain text sequence. Like MethVisual QUMA uses a pairwise alignment approach. This means that each clone sequence is aligned with the reference sequence and the alignment of an individual clone sequence is independent of other study clone sequences. As for BIQAnalyzer any AC procedure is not implemented. QUMA, as MethVisual and BIQ Analyzer includes a sequence identity test and a bisulfite conversion quality test. DNA methylation states are displayed as black (methylated) or white (non methylated) circles in a lollipop plot (Figure 5 upper part). The figure demonstrates comparable results as for MethVisual and BIQAnalyzer. In addition, the program offers statistical tests as Mann-Whitney U test and Fisher's exact test and a diagram of comparative methylation; this feature has been implemented in MethVisual as well. The lower part of Figure 5 is comparable with the summary part of Figure 3a (upper part) and displays the relative amount of methylation for each CpG site. The user might decide which kind of display he prefers.

Coming back to the MethVisual display in Figure 3c we show a correspondence analysis plot, that cannot be offered by BIQ Analyzer and QUMA. The plot is very intuitive. For example, CpG 2 is mainly determined by its methylation profile of sequence C which is -going back to the lollipop display in Figure 3a - reasonable. Only sequence C shows a methylation in CpG site 2. Even though features like this can also be observed in the lollipop display, correlated features as CpG site 2 and sequence C are easier to see from the correspondence analysis plot. Also, the relative strength of correlation which is given by the vector lengths and angles is easier to investigate. Overall the two dimensional display captures 88.88% of total inertia (58.21% in the first component and 30.59% in the second).

### DNA methylation data processing: biological data

In order to include an experimental dataset we analyzed a published bisulfite sequence dataset starting with the sequencer output as provided by the authors [37]. Dokun et al. [37] investigated the relationship between SNCG, S100A4, S100A9 and LNC2 gene expression and DNA methylation in bladder cancer. SNCG is 4.7 fold stronger expressed in tumor culture and SNCG down regulation is associated with hypermethylation. The results also indicate that the SNCG methylation pattern is cell type specific [37]. We analyzed this bisulfite sequencing data using the MethVisual pipeline. All processing steps like alignments, quality control were done automatically without any manual intervention. All bisulfite sequences passed the QA and QC procedures of the package, showing high quality in all aspects. The quality controlled bisulfite sequences were analyzed with the methVisual and lollipop figures and clustering analysis are demonstrated below. Sequences reading, data processing and all statistical analysis and graphs took approximately 15 seconds on a Windows 32 bit operating system.

The lollipop plot shows similar results as shown by Dokun et al [37] (additional file 2) apart from two features. The first difference is the number of CpG sites that are aligned to the reference sequence. In the Dokun et al [37] there are 14 CpG sites, whereas methVisual found 16 sites. In fact we processed the data also in BIQAnalyzer and in QUMA and got the same results as with MethVisual. We conclude that there are 16 sites. The second difference is the amount of CpG methylation in three out of five cell lines (RT4, Umuc3 and BFTC909). This is due to the alignment gap penalty. Depending on the usage of the penalty, especially in case of Ns appearing in the sequence, the result differs slightly.

Since the dataset is obtained from different cell cultures it is of interest to see whether the single bisulfite sequences can be clustered into subsets. Clustering the data, the hypermethylated BFTC909 and Umuc3 bisulfite sequences are clearly clustered together. Also the hypomethylated bisulfite sequences HT1376 and VmCub1 are classified together (additional file 3). The RT4 bisulfite sequences are the only ones which are divided between the hypo- and hypermethylated classes. That can be explained by looking at the RT4 methylation pattern in the lollipop plot, where the two bisulfite sequences are poorly methylated and the other two are non methylated over all CpG sites.

## Concluding remarks

The visualization and exploratory statistical analysis of genome wide DNA methylation profiles is a fundamental step for the investigation of the regulatory impact of epigenetic processes. Currently bisulfite sequencing serves as a gold standard for DNA methylation profiling. We have shown that MethVisual comprises processing of non high throughput DNA methylation data and furthermore allows for several visualization and basic statistical application steps. Existing tools [16-18] - even though not in R/Bioconductor - show few of these features, thus MethVisual is the first comprehensive DNA methylation processing and analysis tool in R. In this work we describe the package and demonstrate the workflow. We compare alignment and quality check with existing tools (BIQAnalyzer [17] and QUMA [16]) based on the dataset that has been published along with BIQAnalyzer [17] and a biological dataset [37]. We show in a very simple case how intuitive visualization works. We point out that the tool can be used in a straightforward way to analyze high throughput DNA methylation data and expression data integratively by combining existing packages within Bioconductor. High throughput DNA methylation data is at an early stage of production. In the future the package might be broadened to allow high throughput DNA methylation data as derived from second generation sequencing to be processed efficiently within MethVisual.

## Competing interests

The authors declare that they have no competing interests.

## Authors' contributions

CS designed the project, selected the data and wrote the manuscript. AZ programmed the package and processed and analyzed the data. All authors have read and approved the final manuscript.

## Supplementary Material

Additional file 1 **Comparison of BIQAnalyzer and methVisual data processing**. Click here for file

Additional file 2 **Lollipop display of SNCG dataset**. Click here for file

Additional file 3 **Biclustering diagram of SNCG dataset generated by MethVisual R package**. On the y axis bisulfite sequence names are shown. On the x axis CpG positions are displayed numbered according to their relative position on the reference sequence. The clustering is visualized by showing a dendrogram on the upper left side according CpG positioning and bisulfite sequences. The red colored squares mark non methylated CpG sites while the light colored squares mark methylated sites. Click here for file
