# Supplementary material for: MethVisual - visualization and exploratory statistical analysis of DNA methylation profiles from bisulfite sequencing
Source: BMC Res Notes. 2010 Dec 15;3:337. doi: 10.1186/1756-0500-3-337 (PMC3012040; doi:10.1186/1756-0500-3-337)
Supplement: Additional file 1 — Comparison of BIQAnalyzer and methVisual data processing. [file 1756-0500-3-337-S1.DOC]

Table1: Comparison of BIQAnalyzer and methVisual data processing

| Clone name | Identity BiQ | Identity methVisual | Conversion  BiQ | Conversion  methVisual | Alignment  BiQ | Alignment  methVisual |
| --- | --- | --- | --- | --- | --- | --- |
| seqA | 0.91 | 0.78 | 1.0 | 1.0 | normal | normal |
| seqB | 0.99 | 0.99 | 0.96 | 0.96 | normal | normal |
| seqC | 1.0 | 1.0 | 0.99 | 0.99 | Reverse-complement | Reverse-complement |
| seqD | 1.0 | 1.0 | 0.90 | 0.90 | normal | normal |
| seqE | 1.0 | 0.94 | 0.96 | 0.96 | normal | normal |
| seqF | 1.0 | 1.0 | 0.88 | 0.88 | normal | normal |
| seqG | 1.0 | 1.0 | 0.96 | 0.96 | normal | normal |
| seqH | 0.99 | 0.99 | 1.0 | 1.0 | normal | normal |
| seqI | 1.0 | 1.0 | 1.0 | 1.0 | normal | normal |
| seqJ | 0.77 | 0.65 | 0.91 | 0.91 | normal | normal |
