# Supplementary figures and images for: MethVisual - visualization and exploratory statistical analysis of DNA methylation profiles from bisulfite sequencing
Source: BMC Res Notes. 2010 Dec 15;3:337. doi: 10.1186/1756-0500-3-337 (PMC3012040; doi:10.1186/1756-0500-3-337)

MethVisual Lollipop Display with equidistant CpG sites


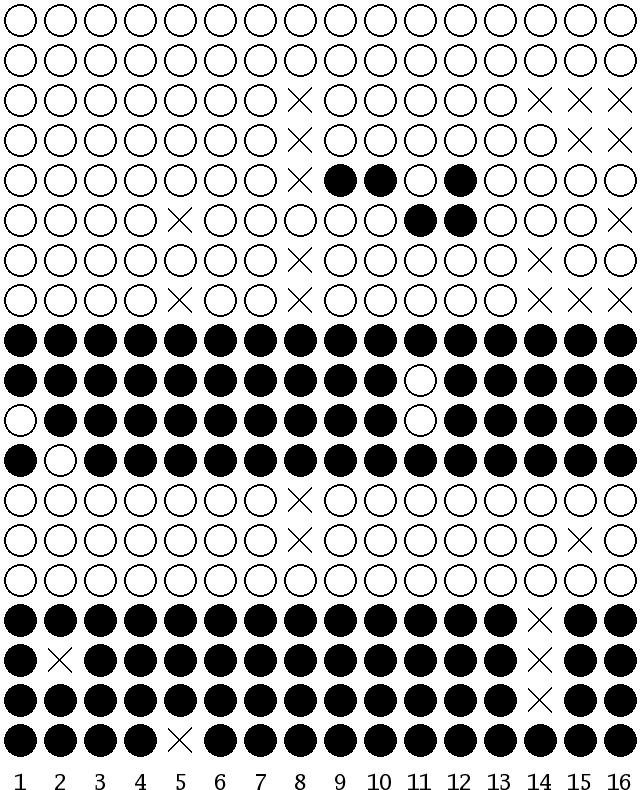


QUMA Display


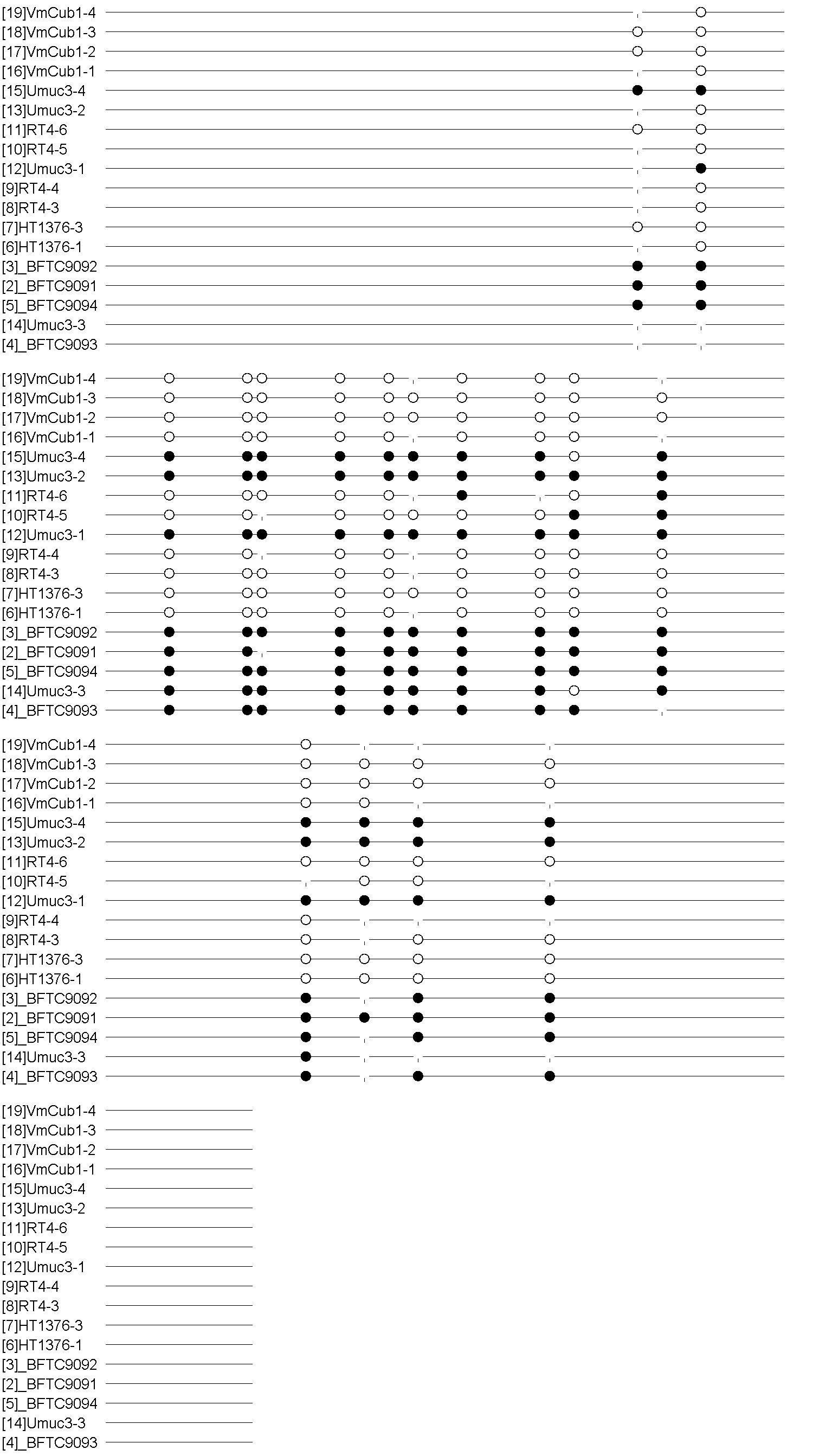


BIQAnalyzer Display

Supplement: Additional file 2 — Lollipop display of SNCG dataset. [file 1756-0500-3-337-S2.DOCX]

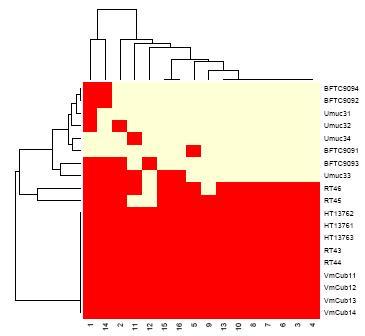

Supplement: Additional file 3 — Biclustering diagram of SNCG dataset generated by MethVisual R package. On the y axis bisulfite sequence names are shown. On the x axis CpG positions are displayed numbered according to their relative position on the reference sequence. The clustering is visualized by showing a dendrogram on the upper left side according CpG positioning and bisulfite sequences. The red colored squares mark non methylated CpG sites while the light colored squares mark methylated sites. [file 1756-0500-3-337-S3.DOCX]
